# Antagonistic and cooperative AGO2-PUM interactions in regulating mRNAs

**DOI:** 10.1038/s41598-018-33596-4

**Published:** 2018-10-17

**Authors:** Erin L. Sternburg, Jason A. Estep, Daniel K. Nguyen, Yahui Li, Fedor V. Karginov

**Affiliations:** Department of Molecular, Cell and Systems Biology, Institute for Integrative Genome Biology, University of California at Riverside, Riverside, CA 92521 USA

**Keywords:** Argonaute (AGO2), Pumice, AGO2 Binding, Pumilio Proteins, Dicer-deficient Cells, RNA, RNA decay

## Abstract

Approximately 1500 RNA-binding proteins (RBPs) profoundly impact mammalian cellular function by controlling distinct sets of transcripts, often using sequence-specific binding to 3′ untranslated regions (UTRs) to regulate mRNA stability and translation. Aside from their individual effects, higher-order combinatorial interactions between RBPs on specific mRNAs have been proposed to underpin the regulatory network. To assess the extent of such co-regulatory control, we took a global experimental approach followed by targeted validation to examine interactions between two well-characterized and highly conserved RBPs, Argonaute2 (AGO2) and Pumilio (PUM1 and PUM2). Transcriptome-wide changes in AGO2-mRNA binding upon PUM knockdown were quantified by CLIP-seq, and the presence of PUM binding on the same 3′UTR corresponded with cooperative and antagonistic effects on AGO2 occupancy. In addition, PUM binding sites that overlap with AGO2 showed differential, weakened binding profiles upon abrogation of AGO2 association, indicative of cooperative interactions. In luciferase reporter validation of candidate 3′UTR sites where AGO2 and PUM colocalized, three sites were identified to host antagonistic interactions, where PUM counteracts miRNA-guided repression. Interestingly, the binding sites for the two proteins are too far for potential antagonism due to steric hindrance, suggesting an alternate mechanism. Our data experimentally confirms the combinatorial regulatory model and indicates that the mostly repressive PUM proteins can change their behavior in a context-dependent manner. Overall, the approach underscores the importance of further elucidation of complex interactions between RBPs and their transcriptome-wide extent.

## Introduction

Post-transcriptional control of gene expression is central to a wide range of cellular processes, ensuring proper cell homeostasis. In addition, it allows for rapid alterations in gene expression, which are necessary for correct developmental transitions, as well as response to environmental changes. The regulation is mainly accomplished by RNA-binding proteins (RBPs) and microRNAs (miRNAs), both of which target mature messenger RNAs by binding to defined sites in the 3′ untranslated region (UTR). Often, these specific factors serve as the target recognition components of larger complexes, which recruit additional, nonspecific factors to stabilize or repress target mRNA expression. Furthermore, the presence of multiple regulatory complexes is thought to have a combinatorial effect on the mRNA. However, while much is known about the individual effects of RBPs and microRNAs, how interactions between the complexes affect downstream gene expression remains to be fully understood.

As a major part of this regulatory model, miRNAs in stable association with Argonaute (AGO) proteins recruit repressive complexes to target mRNA sites through perfect complementarity within the miRNA “seed” (nucleotides 2–8) and imperfect base-pairing throughout the rest of the 22–23 nt miRNA^1–3^. The Argonaute-containing RNA-induced silencing complex (RISC) engages decapping/deadenylation enzymes to cause mRNA destabilization, or leads to translational inhibition by a mechanism that is still under investigation^4–6^. MicroRNA-guided silencing is expansive across the transcriptome, regulating more than 60% of all genes at different developmental and physiological states^7^.

Pumilio proteins are a prototypical group of predominantly repressive sequence-specific mRNA regulators which are conserved across eukaryotes and participate in similar regulatory processes in many species^8–10^. The family is classified by the Pumilio Homology domain (Pum-HD), which adopts an arc-shaped fold capable of directly binding RNA^10–12^. The canonical Pum-HD is composed of eight alpha-helical repeats, each recognizing one nucleotide of its binding motif, 5′-UGUAnAUA^13,14^. In *Drosophila*, Pumilio proteins regulate embryonic development^15–18^, neuronal function^19–21^, and germline development/maintenance, with the latter function also observed in *C. elegans*^9,22–25^.

In mammals, there are two Pumilio proteins, PUM1 and PUM2, which carry out similar functions with some redundancy and specificity. The paralogs are nearly identical in the Pum-HD (89% in human), have an apparently indistinguishable RNA binding motif^13,26–28^, and share a fairly ubiquitous profile of expression^29,30^. Pum1,2 double knockout mice are inviable^27^, while single knockouts demonstrate roles in spermatogenesis and primordial folliculogenesis^31–33^. Similarly, individual roles for Pum2^34–36^ and Pum1^37^ in mammalian neuronal function have been shown, while defects of the neural-specific double knockout are more profound^27^. Consistent with their expression patterns, the proteins are likely to have functions in broader tissues, as cell-based assays show roles in genome stability and cell cycle regulation^38–40^. In reporter assays, the two proteins demonstrate functional redundancy, since depletion of both factors is necessary to abrogate their repressive effects^39,41^. However, Pumilio proteins appear to bind substantially distinct sets of transcripts *in vivo*^26,27^.

Pumilio proteins can function as repressive factors through mRNA destabilization and translational inhibition. Messenger RNA decay is accomplished by recruiting deadenylation/decapping complexes and poly(A) binding protein (PABP) antagonism^41–43^, resulting in a decrease of target mRNA levels^13,38,44^. Although the full mechanism of translational inhibition is still unknown, interference with translation elongation or termination, and competition with eIF4E have been proposed^45–47^. However, Pumilio proteins have also been documented to have stabilizing effects on target mRNAs across eukaryotes^34,44,48–51^. The mechanisms underlying these biologically relevant effects appear to be case-specific and may involve interaction with additional factors and/or changes in 3′UTR secondary structure upon Pumilio binding.

Previous studies provide several examples of interactions between Argonaute and Pumilio in altering gene expression. The two proteins have been shown to act cooperatively to regulate the CDKN1B mRNA, where PUM binding increases AGO binding by causing an mRNA secondary structure switch^39^. A cooperative interaction is also observed on the E2F3 transcript, where PUM binding leads to increased AGO recruitment. Here, alternative polyadenylation can eliminate PUM sites in the 3′UTR, a strategy utilized by cancer cells to escape cell cycle regulation^40^. In addition, transcriptome-wide studies have identified evolutionary and functional evidence of interaction: it has been shown that predicted miRNA binding sites are enriched in the vicinity of PUM sites, and that co-occurrence of PUM and miRNA sites in stem loops or sites of low accessibility correlates with repression of the mRNA^26,52–55^. However, the global extent of the impact of PUM on AGO2 binding and vice versa, as well as the functional consequences of such interactions, has not been experimentally examined with specific site resolution. In the present study, the RBPs’ mRNA occupancy profiles were quantified by CLIP-seq as a function of their partner’s presence, identifying both cooperative and antagonistic effects of UTR co-occupancy and site overlap on binding. Additionally, the data defined differences and similarities in PUM1 and PUM2 interactions with AGO2, providing information on the paralogs’ roles. Finally, individual validation experiments confirmed co-regulatory expression control by PUM and AGO for a subset of identified sites.

## Results

### Determination of AGO2, PUM1 and PUM2 binding profiles and their inter-dependencies by CLIP-seq

The individual binding behavior of Ago and Pum homologs on mammalian mRNAs has been examined, but their effect on each other’s binding and function is largely undetermined transcriptome-wide. Since the human Argonaute homologs AGO1–3 associate with similar sets of miRNAs^56,57^, mRNAs^28^, and proteins^58^, and AGO4 is typically poorly expressed, our analysis focused on the more abundant AGO2 homolog. We have previously used a reciprocal perturbation approach to assess the global AGO2-HuR interactions by quantitative CLIP-seq.^59^. To further investigate potential AGO2-PUM co-regulatory interactions, crosslinking and immunoprecipitation followed by sequencing (CLIP-seq) experiments were performed for the endogenous PUM1, PUM2 and AGO2 proteins in 293 cells and derivatives (Supplementary File 1). For each protein, binding profiles were quantified under multiple conditions. To determine if PUM proteins impact AGO2 binding behavior, AGO2 CLIP-seq was carried out in conditions of PUM1 or PUM2 knockdown with two distinct siRNAs each (average 82–91% knockdown, Fig. S1), and compared to control siRNA knockdowns. Three biological replicates of PUM1 KD and four replicates of PUM2 KD were performed, resulting in 2187553 and 2565487 PCR-collapsed read counts, respectively. Conversely, the impact of AGO association with mRNAs on PUM binding was assessed by PUM1 and PUM2 CLIP-seq in wildtype cells and two independent DICER-deficient clones^60^. Cells lacking DICER cannot produce mature canonical miRNAs and are used to abolish miRNA-guided targeting of the four AGO proteins to mRNAs. After sequencing, filtering and processing, 3028 AGO2, 9727 PUM1 and 4988 PUM2 sites in 3′UTRs were identified in these datasets. To verify the accuracy of the binding sites, comparisons were made to a previously reported PUM2 PAR-CLIP dataset^28^ and several AGO2 and AGO1 HITS-CLIP, PAR-CLIP and CLASH datasets^28,61–63^ obtained from starBase^64^. The number of overlapping peaks with each set were computed. Similarly, the overlaps with each set in 100 control experiments, where the positions of peaks in our data were randomized on the same UTR, were used to calculate the mean and standard deviation of the background overlap expectation. Z scores of the actual overlap numbers were calculated to be 11.9–33.9 for PUM1 and PUM2 overlaps with previous data, and ranged from 6.4 to 51.9 for AGO2 overlaps, indicating very significant agreement between the datasets. Additionally, searches for miRNA seed site complements and PUM sequence motifs were performed (see below).

### Distinct and similar binding characteristics of PUM1 and PUM2

**Figure 1:**
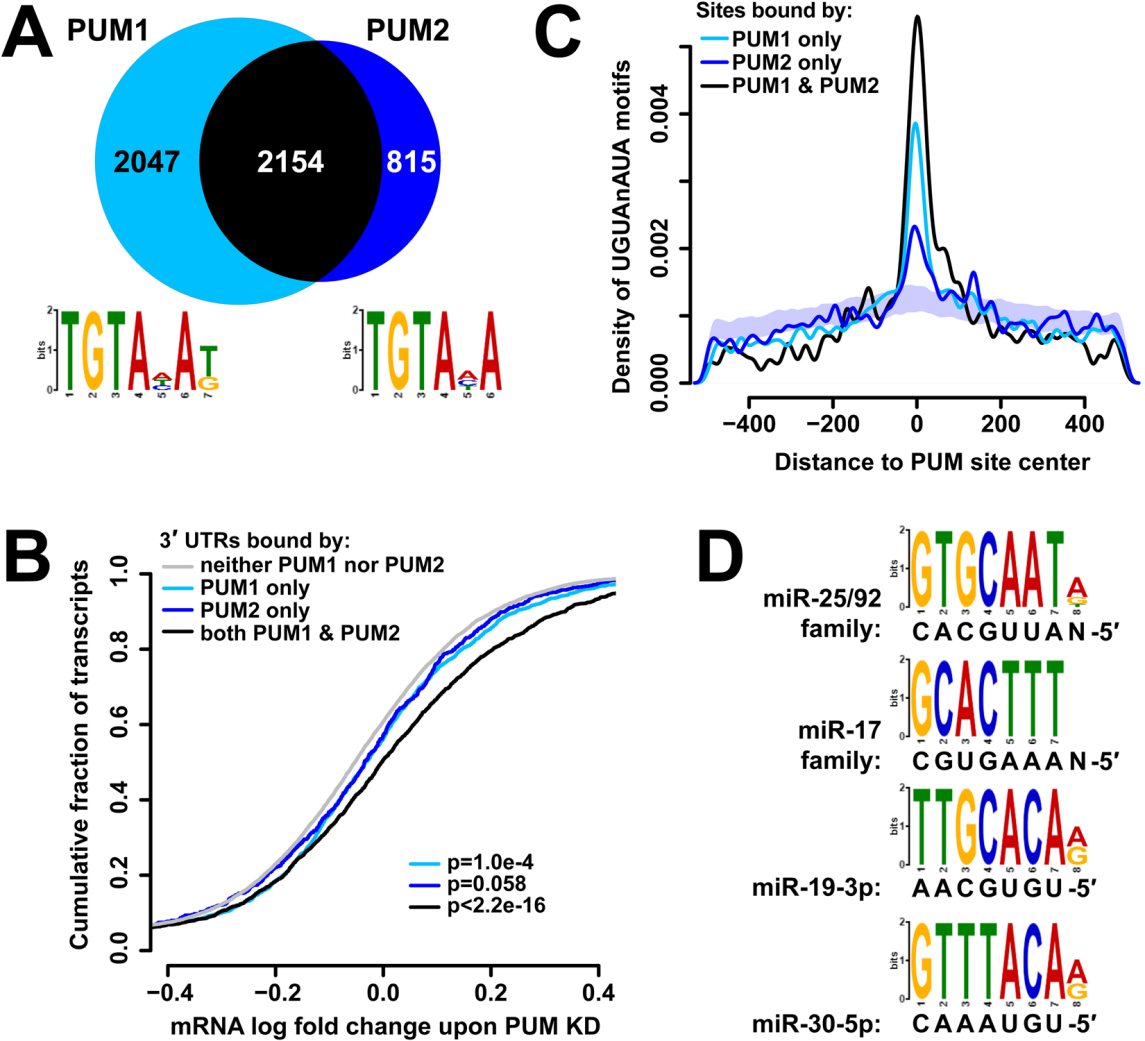
PUM1 and PUM2 have distinct and similar binding characteristics. (**A**) Number of transcripts with 3′UTRs bound by PUM1 (light blue), PUM2 (dark blue), or both (black). Motif logos at the bottom depict the top-scoring enriched motif identified by DREME for the PUM1 and PUM2 sites, consistent with the previously determined PUM motif. (**B**) Cumulative distribution plot of mRNA level log fold change upon PUM knockdown^52^ for populations of transcripts with 3′UTRs bound by PUM1 (light blue), PUM2 (dark blue), both (black), or neither (gray). p-values correspond to a Kolmogorov-Smirnov test of each transcript set against the rest of the transcriptome. (**C**) Density of PUM motifs near PUM1 (light blue), PUM2 (dark blue), or overlapping (black) PUM sites. The shaded area represents a mean +/− 1 SD interval of 100 control densities where PUM motif locations were randomized within each 3′UTR, and distances to PUM2-only peaks computed. (**D**) DREME differential motif enrichment between PUM2 and PUM1 bound sequences show an enrichment of miRNA seed complements within the PUM2 sites.

Human PUM1 and PUM2 share 69% identity / 74% similarity along their entire length, and their mouse orthologs have partially redundant but distinct functions in regulating the cell cycle and developmental processes^27,31,33^. However, their transcriptome-wide binding repertoires have not been examined and contrasted in detail. To this end, we compared the CLIP-sequencing datasets collected for PUM1 and PUM2. At the level of transcripts, PUM2 bound to 2969 3′UTRs, a large majority of which, 2154, were also bound by PUM1, consistent with a partially redundant role for PUM2. PUM1 occupied a broader set of transcripts, with an approximately equal number of 3′UTRs not bound by PUM2 (Fig. 1A). *De novo* motif enrichment analysis^65^ in sites bound by PUM1 and PUM2 (Fig. 1A, DREME E-values of 5.6e^−200^ and 2.5e^−64^, respectively) revealed versions of the previously determined PUM motif as the top identified sequences^10–12^.

To assess the effects of PUM binding on mRNA levels, we compared the distributions of mRNA abundance log fold changes upon knockdown of both PUM1 and PUM2^52^ for transcript classes with various combinations of PUM1 and PUM2 binding in the 3′UTR (Fig. 1B). Transcripts bound by PUM1 or PUM2 alone showed stabilization of mRNA levels, albeit below statistical significance for PUM2 (p = 0.058, Kolmogorov-Smirnov (KS) test). This suggests that such uniquely bound populations of transcripts experience changes in abundance, supporting a non-redundant function at least for PUM1. However, transcripts bound by both PUM1 and PUM2 showed a substantially larger increase in abundance upon PUM knockdown, indicating that jointly targeted transcripts are under more regulation. Furthermore, the extent of PUM1 and PUM2 CLIP signal on 3′UTRs correlated with changes in mRNA levels upon PUM KD (Fig. S2), aided by the elimination of PCR bias in read counts through the use of random barcodes in the ligated CLIP adaptor^66^. Similar observations have been made for the correlation of AGO CLIP strength with target repression^67^, indicating that CLIP data can quantitatively reflect downstream effects on the transcripts.

At the level of individual binding sites, the two proteins exhibited an even lower extent of overlap, with 1226 of 9727 PUM1 peaks and 1233 of 4988 PUM2 peaks overlapping each other (Fig. S3). The PUM1- and PUM2-only populations of peaks were still enriched in the canonical PUM motif sequences (Fig. 1C), suggesting that some of the identified paralog-only sites possess functionality and binding specificity. Interestingly, a distinguishing sequence feature emerges from differential motif enrichment between PUM1 and PUM2 binding sites: sequences matching the seed complements of four abundant miRNAs are enriched in PUM2 relative to PUM1 sites (Fig. 1D). Such an enrichment suggests PUM2 and AGO binding sites are often found in close proximity and have the potential for interaction.

### AGO2 binding is affected by PUM presence on the same 3′UTR, suggesting direct interactions

**Figure 2:**
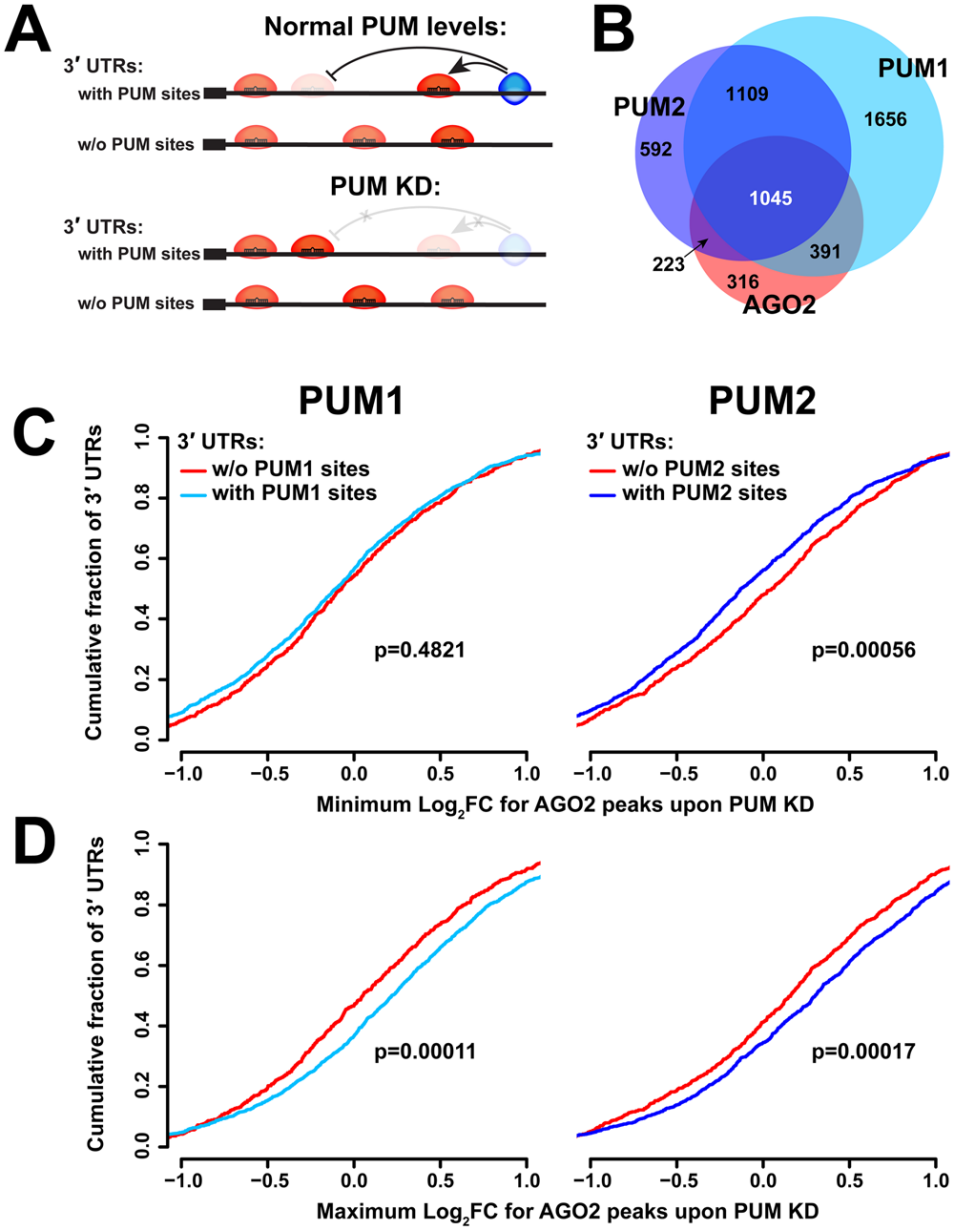
PUM and AGO2 occupy similar transcript populations, and AGO2 binding is affected by PUM presence on the same UTR. (**A**) Schematic of potential interactions between AGO2 and the PUM proteins. (**B**) Number of 3′UTRs bound by any combination of PUM1 (light blue), PUM2 (dark blue), and AGO2 (red). (**C**) Cumulative distribution plot of minimum log fold change for AGO2 peaks on 3′UTRs with and without Pumilio proteins. (**D**) Cumulative distribution plot of maximum log fold change for AGO2 peaks on 3′UTRs with and without Pumilio proteins.

AGO and PUM proteins have been shown to interact cooperatively on the CDKN1B and E2F3 transcripts^39,40^, and previous transcriptome-wide analyses indicate that predicted miRNA seeds and PUM motifs in 3′UTRs are enriched in each other’s vicinity, often in self-complementary secondary structures, and such arrangements lead to faster transcript decay^26,52,54,68^. To further understand interactions between AGO and PUM, the extent of AGO2 binding at 3′UTRs (quantified by CLIP-seq) were compared between control and PUM knockdown conditions and normalized to the changes in mRNA levels upon PUM1 and PUM2 knockdown reported in^52^. Our initial analysis aimed to determine if AGO2 binding is influenced by the presence or absence of PUM on the same 3′UTR (Fig. 2A). A majority of transcripts bound by AGO2 were also occupied by one or both of the PUM proteins (Fig. 2B), indicating a large potential for interactions.

Each UTR can possess multiple AGO2 sites with individual responses to the presence of PUM (i.e. log fold changes in CLIP-seq signal upon PUM KD after mRNA level correction; LFCs), and a minority of all sites are expected to interact with PUM. Thus, to examine transcriptome-wide evidence of co-regulation, we hypothesized that the AGO2 sites with the greatest changes for each transcript (the sites with the minimal LFC and maximal LFC within each UTR) are the most likely candidates for interaction, and the analysis focused on such sites. UTRs were then pooled into two categories for comparison: those bound by both AGO2 and PUM1 or PUM2 (therefore potentially interacting on the 3′UTR per se), and a control group bound only by AGO2 and not the corresponding PUM, thus incapable of an interaction on the UTR, (i.e. representing secondary, background effects). When examining AGO2 sites with the minimal LFC upon PUM2 knockdown within each UTR, sites on transcripts co-occupied by PUM2 showed lower minimal LFCs, compared to peaks on transcripts bound by AGO2 alone (Fig. 2C). This difference suggests the presence of cooperative binding interactions between PUM2 and AGO2, since co-occupancy with PUM2 corresponds with more AGO2 dissociation upon PUM2 KD. Interestingly, such a relationship was not observed between PUM1 and AGO2. Conversely, the maximum log fold change of AGO2 binding upon PUM knockdown is higher on transcripts that are co-occupied by PUM proteins (stronger association), compared to transcripts bound by AGO2 alone (Fig. 2D), consistent with AGO-PUM antagonism. In summary, these results indicate that AGO2 binding on PUM-bound 3′UTRs is differentially impacted by PUM knockdown, strongly suggesting interactions between the proteins on the same 3′UTR.

### AGO2-PUM site co-occupancy affects PUM binding

**Figure 3:**
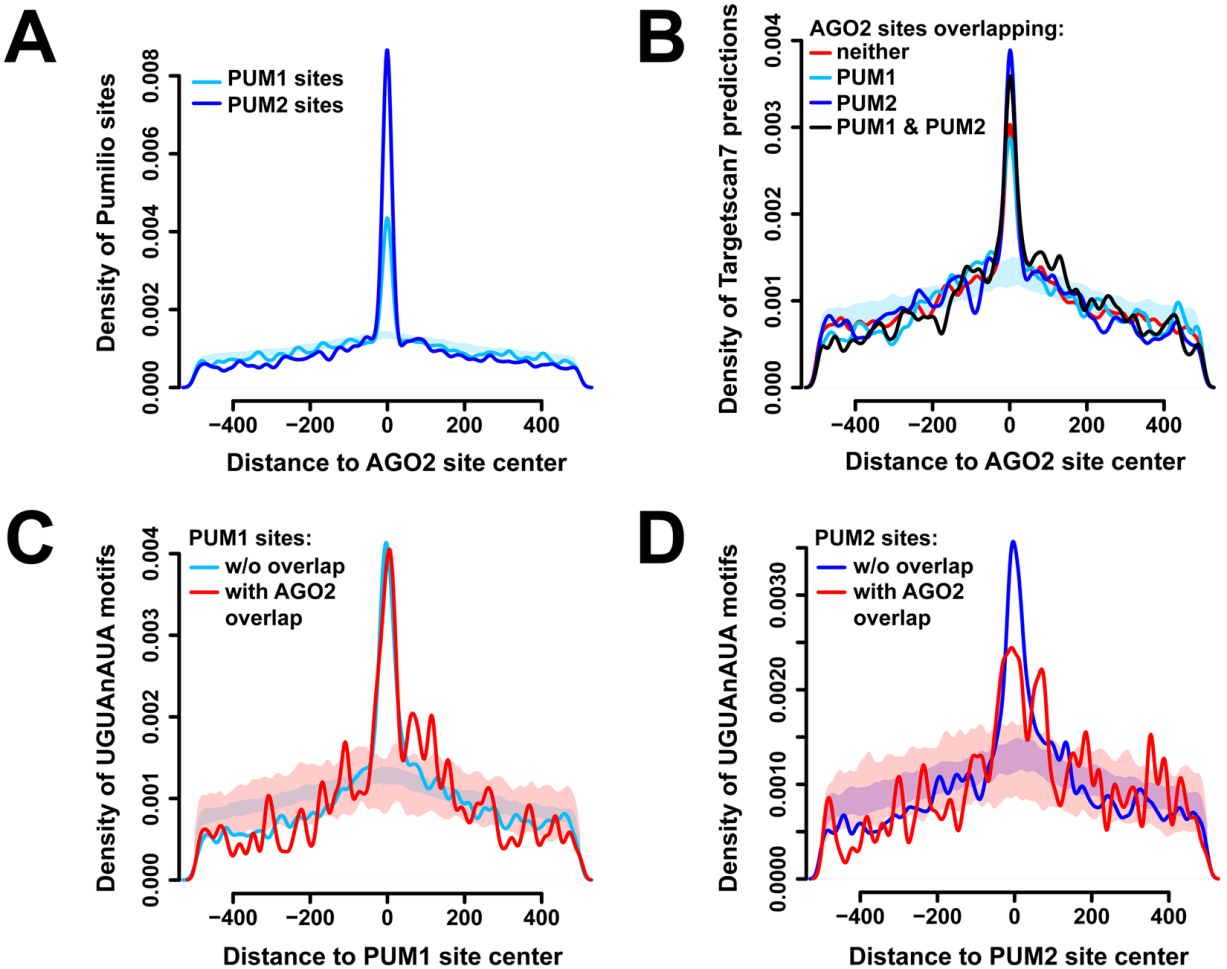
AGO2 and PUM proteins show overlap at the binding site level. (**A**) Density of PUM1 (light blue) and PUM2 (dark blue) binding sites surrounding AGO2 binding sites. The shaded area represents a mean +/− 1 SD interval of 100 control densities where PUM1 site locations were randomized within each 3′UTR, and distances to AGO2 peaks computed. (**B**) Density of Targetscan7 predictions surrounding AGO2 peaks, separated by sites that overlap with PUM1 (light blue), PUM2 (dark blue), both (black), or neither (red). The shaded area represents randomized PUM1-only controls as above. (**C,D**) Density of PUM motifs surrounding PUM1 (**C**) and PUM2 (**D**) sites, separated by populations that do (red) or do not (blue) overlap with AGO2. The shaded areas represent randomized controls for the corresponding populations.

Next, the extent of overlap between AGO2 and PUM sites, and its potential effects on protein binding, were analyzed. Of the 3028 AGO2 sites across all 3′UTRs, a significant fraction − 794 and 800 sites - overlapped with PUM1 or PUM2, respectively (Fig. S4). Notably, the number of AGO2/PUM2 overlaps is larger, despite the roughly two-fold fewer total PUM2 vs. PUM1 sites. The extent of co-occupancy is also evident in positional enrichment of PUM1 and PUM2 around AGO2 sites (Fig. 3A), with PUM2 again being more dominant. These results are consistent with the differential enrichment of miRNA seed complements observed in PUM2 sites relative to PUM1 (Fig. 1D). To ensure that the overlapping peaks represent true AGO2 and PUM populations and not procedural CLIP artifacts (such as cross-linking hot spots), the density of Targetscan7-predicted miRNA seed complements^69^ within AGO2 peaks were examined, which demonstrated enrichment regardless of whether they overlap with the PUM proteins (Fig. 3B). Additionally, the experimentally determined AGO2 sites were found to be centered on the predicted miRNA seed occurrences (Fig. S5). Similarly, PUM binding sites show enrichment of the consensus motif with and without AGO2 overlap (Fig. 3C,D). To assess if specific miRNA families are used in targeting individual vs. PUM-overlapping AGO2 sites, occurrences of each miRNA family seed complement in sites were tallied and compared. Overlapping and non-overlapping AGO2 sites were found to have similar miRNA repertoires (Spearman correlation coefficient of 0.651 and 0.647 for PUM1 and PUM2 overlap, respectively), suggesting that AGO2 sites with PUM overlap do not prefer specific miRNAs.

**Figure 4:**
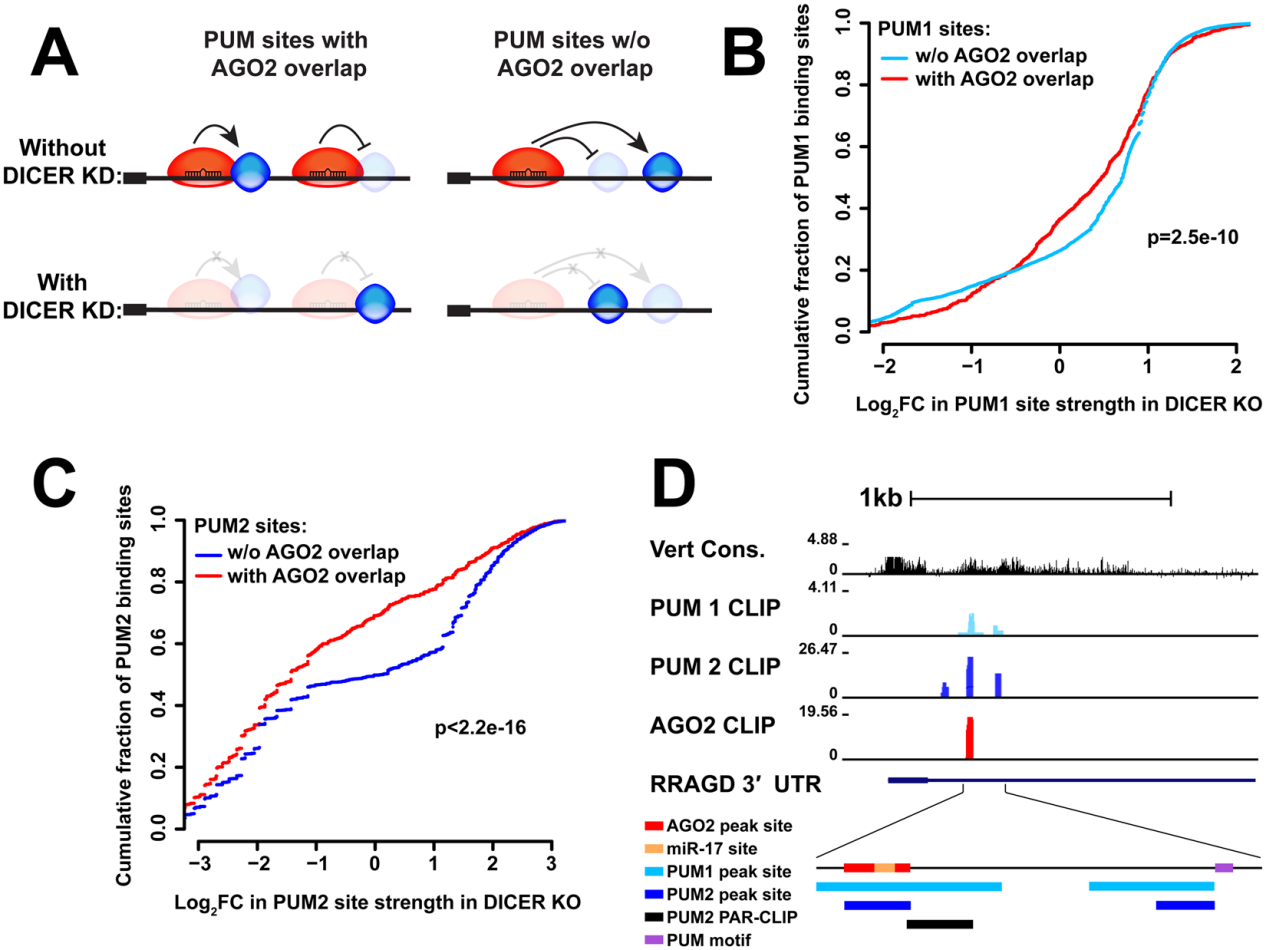
AGO2-PUM site co-occupancy affects PUM binding. (**A**) Schematic of possible interactions between AGO2 and the PUM proteins for PUM sites with and without AGO2 overlap, as well as predicted effect of DICER knockdown in each case. (**B**) Cumulative distribution plots of the log fold change in PUM1 site strength, with (red) and without (light blue) AGO2 overlap. (**C**) Cumulative distribution plots of the log fold change in PUM2 site strength, with (red) and without (dark blue) AGO2 overlap. (**D**) Example UCSC browser view of a site with overlapping PUM1 (light blue), PUM2 (dark blue), and AGO2 (red) binding within the RRAGD 3′UTR. PUM1 and PUM2 binding sites show overlap with an independently generated PUM2 PAR-CLIP and are in close proximity to a PUM motif. The AGO2 binding site contains a predicted miR-17 site.

To determine if overlap with AGO2 affects PUM occupancy, we examined the change in PUM1 and PUM2 binding strength between wildtype and DICER-deficient cells, where miRNA-guided AGO2 association with transcripts is abolished^60^. Comparing the LFCs of PUM binding upon DICER loss among two groups of PUM sites (those with and without AGO2 overlap, Fig. 4A) revealed that both PUM1 and PUM2 binding is differentially weakened at sites of co-occupancy (Fig. 4B,C). In this analysis, PUM2 also exhibited a stronger dependence on AGO2 association than PUM1. Thus, the loss of PUM binding as a result of eliminated AGO2 binding suggests the presence and predominance of cooperative (as opposed to antagonistic) interactions between the two proteins.

### Validation of CLIP-seq candidate sites demonstrates antagonistic PUM-AGO2 interactions

**Figure 5:**
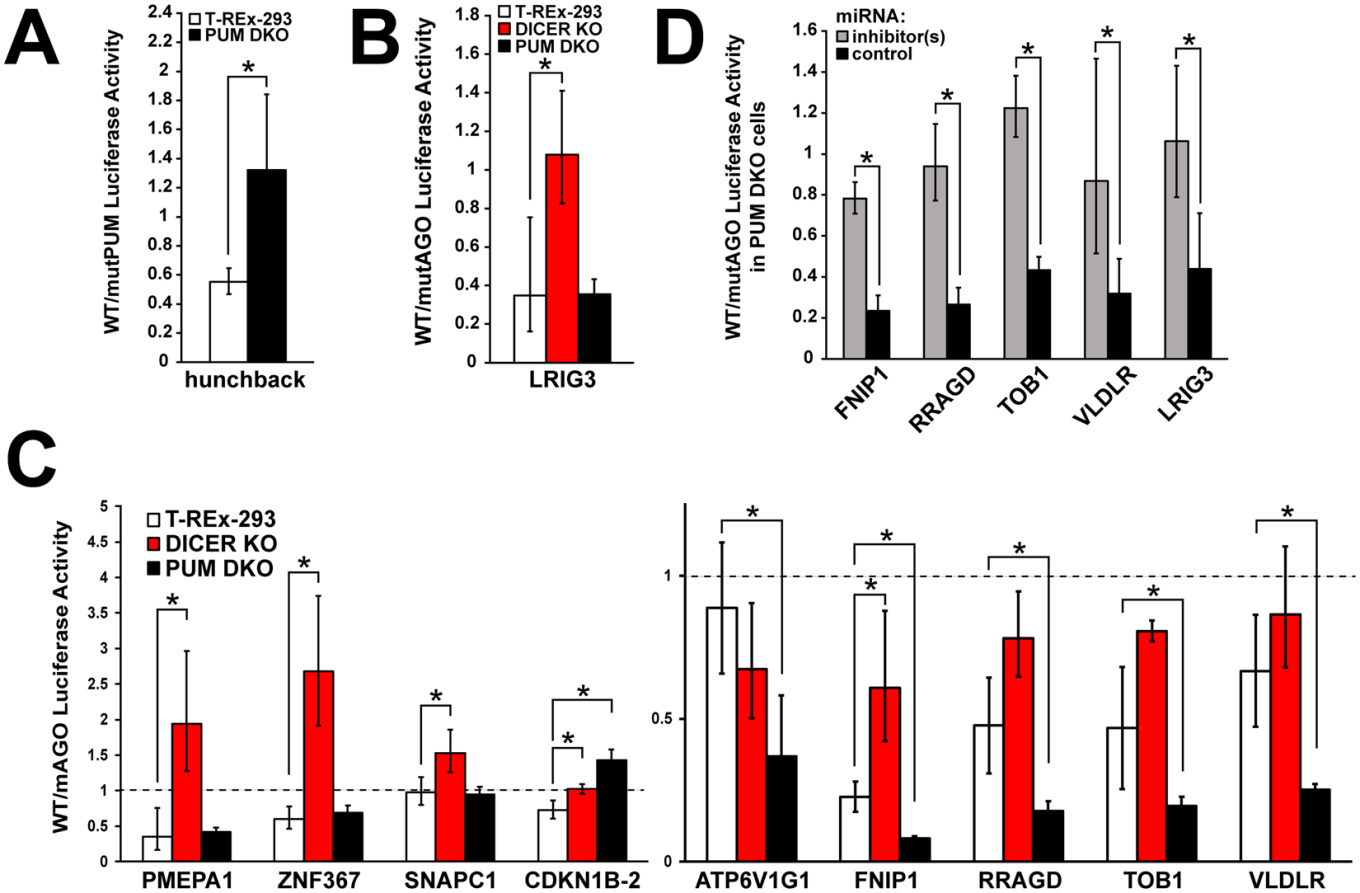
Luciferase reporter assays of candidate sites reveal antagonistic AGO2-PUM interactions. (**A**) A known Pumilio-regulated site from the 3′UTR of the *Drosophila* hunchback mRNA shows loss of regulation in PUM double knockout cells. (**B**) A strong AGO2 site in the LRIG3 3′UTR with a predicted miRNA seed shows loss of repression in DICER knockout, but not PUM double knockout, cells. (**C**) A subset of candidates show miRNA-dependent activity (PMEPA1, ZNF367, SNAPC1) or both miRNA and PUM dependence (CDKN1B-2, ATP6V1G1, FNIP1, RRAGD, TOB1, VLDLR). Candidate sites were tested in three conditions: wildtype T-REx-293, DICER knockout cells, and PUM double knockout cells. (**D**) Sites co-regulated by AGO and PUM sites show dependence on specific miRNAs in PUM double knockout cells. For FNIP1 and VLDLR, WT and mutant AGO peak constructs were used. For RRAGD and TOB1, WT and mutant miRNA seed constructs were used.

Since the transcriptome-wide analysis suggested that overlapping sites may host interactions, candidates were selected to test whether co-regulatory effects on protein expression could be detected. To increase the likelihood of identifying such sites, we picked from the top 20 strongest normalized and unnormalized AGO2 binding sites based on overlap with a PUM2 binding site in our data and with a previously published PUM2 PAR-CLIP dataset^28^, yielding 26 sites. An example UCSC browser shot of a candidate site is shown in Fig. 4D. Sites ranging 76–330 nt in length, including the above elements with 10 nt of flanking sequence, were 4X concatenated and cloned into the 3′UTR of the *Renilla* luciferase reporter gene in the psiCheck2 plasmid with an internal firefly normalization control. In parallel, constructs with a mutated (shuffled) AGO2 site were also generated. The reporters were used to determine the regulatory activity of the AGO site, as the ratio between wildtype and mutant luciferase activity (WT/mut ratio), in three distinct cellular settings. Wildtype T-REx-293 cells reported on the site’s activity in the presence of PUM, and DICER-deficient 293 T derivatives^60^ uncovered the miRNA-targeted contribution to the regulation. In turn, to assess the effect of PUM on the regulation, PUM double knockout (PDKO, Fig. S6) cells were generated using CRISPR-Cas9 combined with homologous recombination^70^. To confirm that PUM has been functionally knocked out, a Renilla luciferase reporter with a 4X concatenated sequence of a known Pumilio-regulated site from the 3′UTR of the *Drosophila* hunchback mRNA was used (Fig. 5A). Similarly, as a control for miRNA-guided regulation, a strong AGO2 site in the LRIG3 3′UTR with a predicted miRNA seed that does not contain a PUM motif was chosen and cloned as a 4X concatamer. Confirming the expectation that the AGO2 site is repressive, the WT/mut ratio was shown to be significantly less than one in T-Rex-293 cells, and the repression was DICER- but not PUM-dependent (Fig. 5B).

When tested in the above settings, the candidate panel demonstrated several regulatory patterns. Candidates that showed no regulation, a stabilizing/repressive site activity that was not dependent on either PUM or DICER, or excessively high protective site activity, (Fig. S7) were not examined further. Two sites (PMEPA1 and ZNF367, Fig. 5C) possessed a DICER-dependent, but PUM-independent repressive activity, representing canonical AGO regulation. The SNAPC1 site showed no regulation in WT and PDKO cells, but stabilization in DICER KO cells, suggesting a miRNA dependence that does not fit the canonical model, or indirect effects (Fig. 5C). Interestingly, our selection criteria, informed by precise AGO2 and PUM binding locations, identified two overlapping sites in the CDKN1B 3′UTR, where a PUM/AGO2 interaction mediated by two miR-221/222 seed sites was previously demonstrated^39^. For the CDKN1B-1 construct, which included one of the previously implicated miR-221/222 seeds under the AGO2 site (Fig. S8), mutation of the site did not reveal any statistically significant differences in regulation between WT and PUM- or DICER-deficient cells (Fig. S8). However, activity at a separate, 3′-proximal CDKN1B-2 site was dependent on both DICER and PUM.

Finally, five sites demonstrated either no effect in WT cells, or a repression that was attenuated in DICER KOs; however, all five became substantially more repressive in PDKOs (Fig. 5C, ATP6V1G1, FNIP1, RRAGD, TOB1, VLDLR). This pattern suggests a previously unobserved antagonistic interaction between PUM and AGO. Since the repressive activity of the AGO site in wildtype cells was minimal for most of the sites, we wanted to confirm whether the increased repression in PDKO cells was directed by the miRNA machinery. To this end, inhibition of specific target miRNAs was performed in PDKO cells for four of the five antagonistic sites, and ratios of WT to AGO site mutant reporter constructs were measured as above (Fig. 5D). ATP6V1G1 was excluded from further analysis because it did not contain a predicted miRNA seed. The increase in repression in the absence of PUM was confirmed to be miRNA dependent (Fig. 5D), indicating that miRNA-guided repression can occur at these sites, but is prevented when Pumilio proteins are present in the cell.

### PUM antagonizes AGO through the predicted Pumilio motif

**Figure 6:**
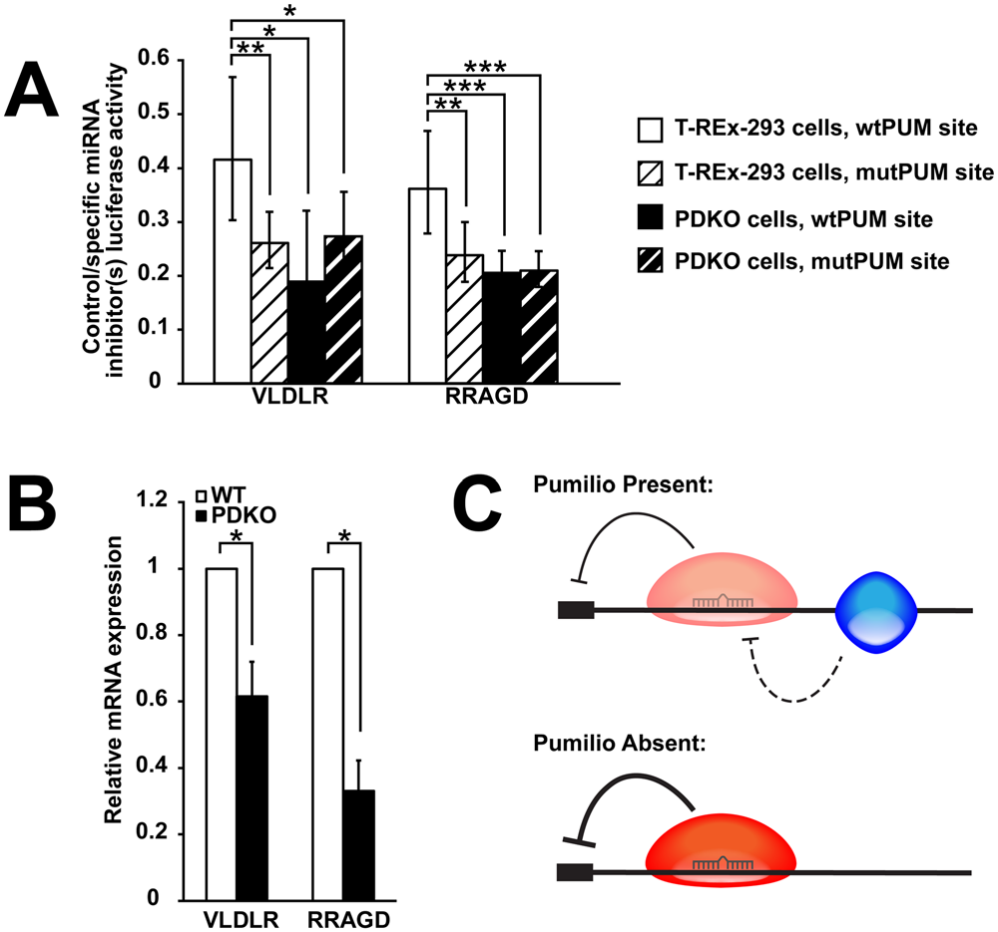
PUM antagonizes AGO through the predicted PUM motif, and PUM double knockout stabilizes endogenous transcripts. (**A**) Sites co-regulated by AGO and PUM show dependencies on specific miRNAs, PUM proteins, and the predicted PUM site. Luciferase reporter constructs co-transfected with control or specific miRNA inhibitors were tested under wildtype (white) and PUM double knockout (black) conditions with WT (solid) or mutant PUM sites (stripes). (**B**) RT-qPCR of endogenous VLDLR and RRAGD transcripts in both wildtype (white) and PUM double knockout (black) cells. (**C**) A model of the antagonistic effect of PUM on AGO2 regulation of VLDLR and RRAGD mRNAs.

Next, we aimed to determine whether the observed antagonism is due to the presence of PUM binding to the predicted motif(s) adjacent to the AGO site, or if this change in AGO activity is due to secondary or trans-regulatory effects. Constructs with mutations in the PUM motifs (perfect and imperfect) instead of the AGO site were generated. miRNA dependence was again determined by comparing luciferase activity under specific and control miRNA inhibitor conditions, while the effects of PUM on the miRNA-dependent activity were then tested under wildtype site, mutant PUM site, and PDKO conditions. Importantly, VLDLR and RRAGD showed equivalent repression whether the protein, its sites, or both were removed (Fig. 6A), indicating that regulation by PUM is only due to PUM’s action at the predicted sites. Activity of the FNIP1 and TOB1 reporters in this assay was inconsistent with such a model (Fig. S9): PUM effects for TOB1 were not significant, while FNIP1 showed increased repression for the mutant site construct that did not reach statistical significance, and a further, significant repression when the protein was removed (Fig. 6A). However, removal of both the PUM protein and site restored FNIP1 miRNA repression to WT levels. One interpretation is that the mutated PUM site also serves as a binding site for a third RBP that cooperatively interacts with AGO2. Overall, the results for VLDLR and RRAGD demonstrate an antagonistic model of interaction between AGO and PUM at overlapping sites.

To determine whether a physical AGO2-PUM interaction could be detected, co-immunoprecipitation experiments were performed. Immunoprecipitation (IP) of AGO2 followed by immunoblot of the two PUM proteins did not show any interaction (Fig. S10A). However, IP of both PUM1 and PUM2 showed a weak interaction with AGO2. For PUM1, additional washes disrupted most of this interaction, and RNase treatment abolished the interaction entirely (Fig. S10B). For PUM2, a weak interaction was detected in the IP (Fig. S10C), but the PUM2-bead complex was lost in subsequent treatments. These results suggest that AGO2 and PUM proteins do not form a stable stoichiometric complex, and may enter into relatively weak and transient interactions. Consistent with these observations, binding of endogenous PUM2 to tagged AGO2 has also been detected by IP-mass spectrometry in one study^71^, but was not identified in another^58^.

Finally, we wanted to confirm that results determined in artificial constructs correspond to effects on endogenous messages. To do so, RT-qPCR was performed to determine mRNA levels of both RRAGD and VLDLR mRNAs in wildtype and PDKO cells. Results show that both transcripts are downregulated in PDKO cells when compared to WT (Fig. 6B). This result is consistent with our reporter constructs, and demonstrates an unexpected, protective activity in conjunction with AGO2, in contrast to Pumilio’s canonical repressive role.

## Discussion

We employed a transcriptome-wide approach to determine the binding profiles and inter-dependencies of AGO2, PUM1, and PUM2 sites on mRNA 3′UTRs. In agreement with the extensive homology between the two PUM proteins, binding sites of both paralogs showed enrichment of a nearly identical motif consistent with the known UGUAnAUA recognition sequence, and there was a significant amount of overlap in the populations of transcripts bound by the proteins. However, PUM1 bound a substantially larger population of transcripts than PUM2 (Fig. 1A). Interestingly, previous studies utilizing a different method (RIP-Chip) or different antibodies in CLIP-seq similarly identified approximately twice as many transcripts associated with PUM1 vs PUM2, with comparable overlap^26,27^, strongly suggesting that these observations reflect their intrinsic binding properties. The greater number of transcripts may be explained by a higher binding affinity (possibly mediated by PUM1’s longer N-terminal extension), or additional specificity determinants associated with PUM2 binding. Transcripts bound exclusively by PUM1 showed a detectable amount of repression, suggesting that PUM1-specific targeting of mRNA populations is biologically relevant (Fig. 1B). Nevertheless, transcripts bound by both paralogs showed substantially greater regulation. Overlap of individual PUM1 and PUM2 binding sites was significantly less common than that of 3′UTRs, with paralog-specific loci still enriched in the binding motif (Fig. 1C). Such loci included peaks from abundant transcripts supported by many individually barcoded ligation event read counts (Fig. S3) indicating that the observed specificity is unlikely to arise from undersampling. It is yet to be determined how PUM proteins can occupy distinct binding sites despite a shared motif and extensive protein homology, although potential interaction partners may be involved. In accord with this model, differential motif discovery between PUM1 and PUM2 sites showed an enrichment of miRNA seeds near PUM2 sites (Fig. 1D), suggesting a greater extent of interaction between PUM2 and the miRNA machinery.

Quantitative examination of AGO2 and PUM CLIP data while manipulating the levels/occupancy of the other factor provided direct transcriptome-wide evidence of interactions between the RBPs at the binding level. The majority of AGO2-bound transcripts were also occupied by one or both of the PUM proteins (Fig. 2B), and changes in PUM protein level lead to changes in AGO2 binding strength on co-occupied 3′UTRs. Here, PUM2 showed a signature of cooperative binding with AGO2, and both factors demonstrated antagonistic interactions (Fig. 2C,D). Further, AGO2 showed significant overlap with the PUM proteins at the binding site level, and PUM binding at such loci was differentially weakened upon elimination of AGO2 association, suggesting an overall predominance of cooperative interactions between the factors. Again, PUM2 demonstrated a stronger effect in this analysis (Fig. 4C) and had a higher degree of overlap with AGO2 sites (Fig. 3A). It should be noted that AGO2 CLIP data was collected under independent PUM1 or PUM2 knockdowns, and not simultaneous reduction of both paralogs. This design allows for a better understanding of the individual interactions of the two Pumilios with AGO2, which are not well studied on a transcriptome-wide scale. However, this design also likely leads to smaller or undetectable interaction signal in cases where the two paralogs have mostly overlapping and redundant roles.

Although the analysis focused on the canonical 3′UTR regulatory region, AGO2, PUM1, and PUM2 sites and their overlap were also observed in other annotation categories. Consistent with the size and function of the coding sequence (CDS) and the 5′UTR, the number of sites for all three proteins was lower in these mRNA regions compared to the 3′UTR, while overlap between AGO and the PUM proteins, was similar or lower (Fig. S4A,B). However, enrichment of the PUM consensus motif was only weakly detected in PUM1 (but not PUM2) CDS sites, (Fig. S4C), and was undetectable in the 5′UTR (data not shown). Consistent with motif enrichment for PUM1 sites in the CDS and its interactions with AGO2 in the 3′UTR (Fig. 4B), LFCs of PUM occupancy upon DICER loss showed that PUM1 binding is differentially weakened at CDS sites of AGO2 co-occupancy (Fig. S11B). At sites where motif enrichment was not found (PUM2 in the CDS, or PUM1,2 in the 5′UTR), no such effects were observed (Fig. S11). These conclusions are consistent with previously reported observations that PUM motifs in CDS, but not 5′UTR regions are enriched^26^ and correlate with mRNA repression by PUM^44^. Additionally, substantial AGO2-PUM site overlap, but no PUM motif enrichment, was detected in the ncRNA category, potentially driven by many spurious interactions of the RBPs with abundant cellular ncRNAs.

Validation of individual overlapping sites using luciferase reporters demonstrated a cooperative interaction on the CDKN1B mRNA, a known target of AGO2-PUM co-regulation^39^, and unexpectedly identified two novel instances where PUM can antagonize AGO at a nearby site (Fig. 5C). For CDKN1B, a 5′-proximal site that partially overlaps the previously characterized miR-221/222 seeds did not exhibit co-regulation in our assay, potentially because further necessary nearby sequence elements were not included in the construct. However, a separate, 3′-proximal overlapping site showed both a PUM and miRNA dependence, consistent with a cooperative interaction, suggesting that AGO2-PUM co-regulation at this 3′UTR occurs through more than one site (Fig. 5C). Lack of a predicted miRNA seed at the second site prevented further examination of its regulation. The identification of antagonistic co-regulation of VLDLR and RRAGD mRNAs prompts a model where binding of PUM near the AGO2 sites leads to attenuation of miRNA-guided repression (Fig. 6C), showing that Pumilio, a normally repressive factor, can take on a stabilizing role in a context-dependent manner, and underscores the flexibility that is imparted on the regulation by combinatorial interactions. These observations are consistent with other studies that have suggested a stabilizing role for the Pumilio proteins^44,49,72,73^ and suggest that further cases of stabilization by PUM may occur through the antagonism of other regulatory RBPs. While the precise mechanism(s) of AGO-PUM antagonism are not understood, involvement of secondary structure rearrangements is a good starting hypothesis that has been previously implicated^39,52,55^. Alternatively, steric clashes may be responsible, although in the VLDLR and RRAGD constructs the PUM motifs and AGO binding sites (narrowed down to the predicted miRNA seeds within) appear to be far enough away from each other that the individual factors would not interfere physically. However, since both proteins recruit large complexes, interference may still be possible. It is possible that additional candidates from the interrogated set involved AGO2-PUM co-regulation, but the required sequence elements were not fully included in the reporter constructs.

In many cases, candidate sites selected for luciferase validation contained multiple Pumilio motifs, including the full consensus sequence (UGUAnAUA) and shorter versions with degeneracies in the more weakly defined 3′ end (UGUAnnUA, UGUAnAnA, or UGUAnAUn). For example, the VLDLR site included three perfect motifs, and all three sites were mutated for testing. Individual site mutants would determine the contributions of each of the sites to the observed antagonistic effect. In contrast, the RRAGD site contained one full and one imperfect PUM motif, which together were sufficient for co-regulation.

While testing of individual targets revealed instances where PUM impacted AGO2 activity, AGO’s reciprocal ability to affect PUM binding was also observed in the CLIP data (Fig. 4B,C). However, the reporter experimental design was not amenable to identify such interactions, since PUM site mutants for the full list of candidates were not independently tested in wildtype and DICER-deficient conditions to isolate the PUM site activity and its dependence on miRNAs. Thus, further testing would likely uncover additional examples and a more diverse interaction profile.

The identified switches from repression to protection for PUM underscores the dynamics of RBP regulation and the necessity to develop a comprehensive, site-specific understanding of their effects on gene expression. For major regulatory proteins such as AGO and PUM, misinterpretation or incomplete information about available regulatory modes can hinder the mechanistic understanding of disease states. For example, previous studies have shown that misregulation of VLDLR leads to many neurodevelopmental disorders, including cerebellar ataxia, mental retardation and disequilibrium syndrome (CAMRQ1) and cerebellar hypoplasia^74–76^. Understanding that this gene is a target for AGO/PUM co-regulation, and knowing that PUM acts to stabilize the transcript in this context, can potentially direct new therapeutic strategies. Similar arguments can be made for RRAGD, which is a component within the amino acid sensing branch of mTORC1 signaling. RRAGD misregulation is correlated with renal and liver cancer prognosis^30^. A better understanding of how protein interactions affect the outcome of gene expression will lead to more precise disease treatment and fewer off-target effects.

Our results expand on a growing number of studies demonstrating interactions of the miRNA machinery on specific mRNAs with PUM and other RBPs, including HuR^77,78^, SFPQ^79^, PTB^80^, and DND1^81^, reviewed in^53,82^, as well as global analyses that identify miRNA-RBP interactions^52,54,83,84^. Overall, the presented pairwise perturbation studies, together with similar efforts on other RBPs^59^ and broader static analyses of RBP co-occupancy on 3′UTRs^68^ will be necessary to uncover the full extent of combinatorial post-transcriptional regulation of mRNAs.

## Materials and Methods

### Cell culture and PUM knockdown

T-REx-293 cells were obtained from Invitrogen, and DICER deficient cells (along with the parental line) were a kind gift from B. Cullen^60^. PUM double knockout cells were generated as previously described^70^. All cells were grown in DMEM (Corning) with 10% fetal bovine serum (Corning) and 10 units/ml of penicillin/streptomycin (Gibco) at 37 °C with 5% CO_2_.

For PUM1 and PUM2 knockdowns, 3 and 4 biological replicates, respectively, of T-REx-293 cells were separately transfected with two distinct siRNAs against either PUM1 or PUM2, or with a GL3.1 siRNA control (Supplementary File 2). TransIT-TKO (Mirus) transfections with 100 nM siRNA were performed for the first PUM1 and PUM2 replicates, and calcium phosphate transfections with 100 nM siRNA were carried out for the later replicates. For each replicate set, three successive transfections 2–3 days apart were performed to get sufficient knockdown. For each replicate/condition, three to six 15-cm plates of cells were collected for the CLIP procedure.

### AGO2, PUM1, and PUM2 HITS-CLIP and data analysis

The AGO2 CLIP protocol was performed as previously described^85^. Mouse anti-AGO2 (Santa Cruz, clone 4F9) antibody was used for AGO2 CLIP and goat anti-PUM1 (Bethyl, A300–201A) and rabbit anti-PUM2 (Bethyl, A300-202A) antibodies were used for PUM1 and PUM2 CLIP, respectively. Two replicate sets of PUM1 CLIP and one set of PUM2 CLIP, were performed in 293 T cells (control), NoDice (2–20) and NoDice (4–25) cells. Libraries were sequenced on an Illumina HiSeq. 2500 instrument with a multiplex of six libraries per lane. Sequencing data was analyzed as previously described^59^, with readcount cutoffs equal to the number of samples in each CLIP set.

### PUM double knockout cell generation

PUM2 single KO cells were generated as described^70^ and were used as the parental line for generating PUM double knockout cells. Single guide RNAs (sgRNAs) targeting exon 4 and exon 15 of the PUM1 gene were each cloned into the pLx330 Cas9-sgRNA expression plasmid (Supplementary File 2). A hygromycin resistance cassette flanked by two 900 nt homology regions within exon 4 and exon 15 were assembled in the pUC-19 vector as described^70^ and used as the template for homology directed repair to replace most of the PUM1 gene with the resistance cassette. Cells were transfected with all three plasmids concurrently, and hygromycin selection was applied after three days. Clonal populations of cells were generated and screened using primers flanking the exon 4 CRISPR-Cas9 cut site (Supplementary File 2). Candidate clones were validated by western blot for absence of both PUM1 and PUM2 using antibodies described above.

### Reporter vector plasmid construction

AGO2/PUM overlapping sites were selected based on criteria outlined in the text. For the purposes of selection of candidates among overlapping sites, AGO2 sites/peak locations and widths were defined from a combined dataset containing AGO2 CLIP data of PUM KDs of the current study, and HuR KDs of Li *et al*.^59^. The top 20 sites sorted by unnormalized or mRNA-level-normalized AGO2 CLIP signal level shared many candidates, and both sets were included in the selection. Candidate sites included the entire AGO2 and PUM peaks of interest, expanded to sequences corresponding to miRNA seeds and PUM motifs within 200 nt, plus 10 nt of flanking sequence. Sites were assembled into 4x concatamers into the 3′UTR of the Renilla luciferase reporter gene contained in the psiCHECK-2 (Promega) plasmid as previously described^59^. In parallel, mutant constructs were generated where the entire AGO2 CLIP peak sequence was shuffled in order to abolish AGO2 binding. All WT and mutant monomer sequences along with primers used for assembly are listed in Supplementary File S3. Hunchback and LRIG3 positive controls were assembled with the same design.

For RRAGD, FNIP1, VLDLR, and TOB1 sites, mutant PUM constructs were generated where TGT was mutated to ACA in the PUM motif. For microRNA inhibitor luciferase reporter experiments with RRAGD and TOB1 sites, mutant miRNA seed constructs were generated where three nucleotides in position 2–7 were mutated. In all cases, constructs were assembled as described above.

### Plasmid transfection and luciferase assays

T-REx-293 and PUM DKO cells were seeded in 96-well plates and transfected in technical triplicate at 70% confluency. For all experiments, WT and mutant plasmids were transfected in parallel. For transfections of the initial 26 candidate set, TransIT-LT1 reagent (Mirus) was used per manufacturer’s instruction to add 10 ng of reporter plasmid to cells. In miRNA inhibitor experiments, calcium phosphate transfection was used to transfect 10 ng of reporter plasmid with 0.75 μM of each inhibitor. Anti-miR-30, anti-miR-25, and the control hairpin inhibitors were manufactured by Dharmacon. Anti-miR-17 family LNA and its control were obtained from Exiqon. Cells were lysed 24 hours after transfection with 20 μl of Passive Lysis Buffer (Promega). Five microliters of cell lysate were used for dual luciferase reporter measurements (Promega). Luciferase substrates were diluted 1:5 in use. Renilla luciferase signal was normalized to the firefly luciferase signal produced from the same plasmid to control for transfection efficiency. For each experiment, at least three biological replicates were performed. Experiments with greater than 50% coefficients of variability between the technical replicates were omitted from downstream analysis. The ratio of normalized WT and mutant luciferase was calculated to determine the effect of site mutation on gene expression. Comparisons between WT and mutant constructs were analyzed by two-tailed paired t-test. Comparisons of WT/mutant ratios between cell conditions were analyzed by two-tailed Welch’s t-test. p-value significance was defined at 0.05.

## Electronic supplementary material

Supplementary Information

Supplementary File S1

Supplementary File S2

Supplementary File S3

## Data Availability

The sequencing raw data and processed binding site data is available at the NCBI Gene Expression Omnibus with accession GSE110520.
