# Supplementary material for: Antagonistic and cooperative AGO2-PUM interactions in regulating mRNAs
Source: Sci Rep. 2018 Oct 17;8:15316. doi: 10.1038/s41598-018-33596-4 (PMC6192998; doi:10.1038/s41598-018-33596-4)
Supplement: Supplementary file 1 — Supplementary Information [file 41598_2018_33596_MOESM1_ESM.pdf]

Antagonistic and cooperative AGO2-PUM interactions in regulating mRNAs

Erin L. Sternburg, Jason A. Estep, Daniel K. Nguyen, Yahui Li, Fedor V. Karginov*

Department of Molecular, Cell and Systems Biology, Institute for Integrative Genome Biology, University of California at Riverside, Riverside, CA 92521

* Corresponding author: karginov@ucr.edu

Supplementary Information: supplementary figure legends and file descriptions

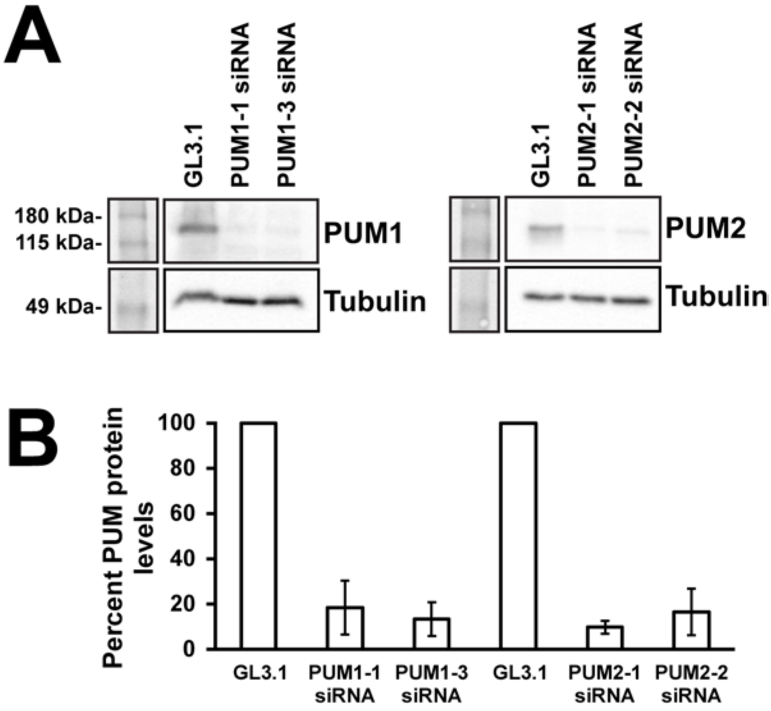

Supplementary Figure 1: PUM1 and PUM2 siRNA knockdown for AGO2-CLIP. **A)** Western blot of PUM1 and PUM2 knockdown with two separate siRNAs each. An siRNA targeting firefly luciferase (GL3.1) was used as a control. **B)** Percent PUM protein after knockdown of PUM1 (n=2) and PUM2 (n=4). Each condition was normalized to total tubulin and then compared to the GL3.1 control.

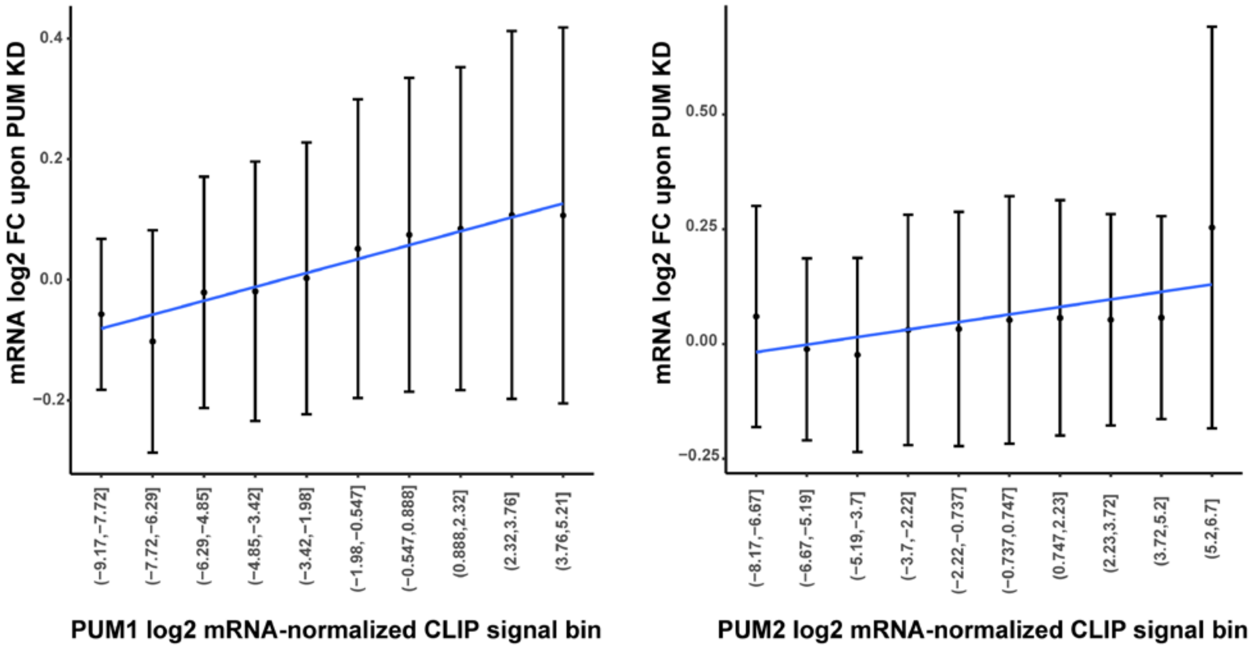

Supplementary Figure 2. Quantitative relationship between PUM CLIP signal strength and effects on mRNA levels. The sum of PUM1 or PUM2 CLIP readcounts for each 3’ UTR, corrected for library depth by DESeq2, normalized to mRNA levels [67], and log2 transformed, were binned. For the transcripts in each bin, the mean mRNA log_2 fold change upon PUM KD [50] was calculated. A) Correlation between mRNA level changes upon PUM KD and PUM1 CLIP signal. Pearson correlation coefficient r = 0.959, p = 1.23 x 10^{-5}. B) Same, for PUM2 CLIP signal; r = 0.658, p = 0.0384.

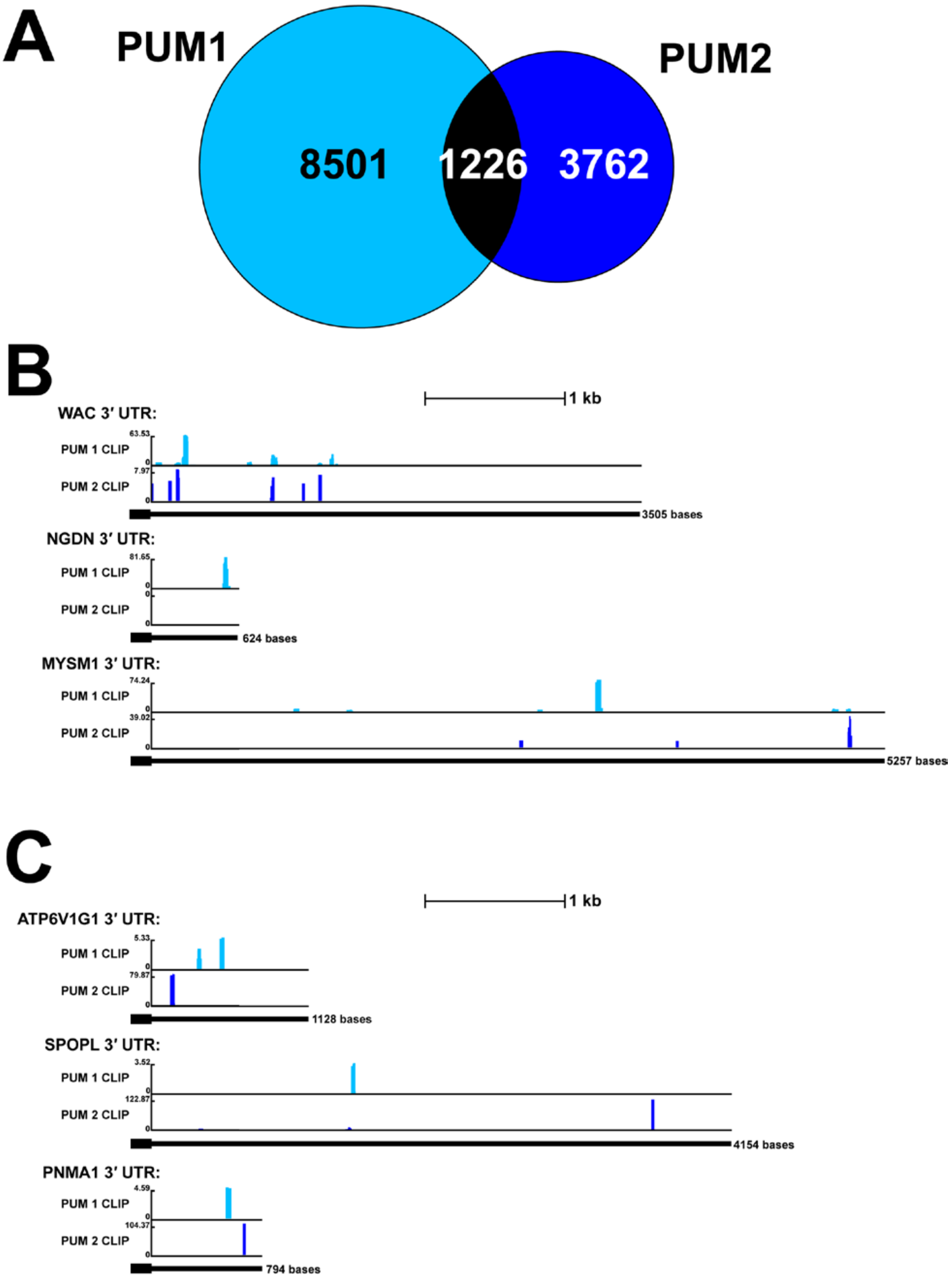

A
PUM1
PUM2
8501
1226
3762
B
1 kb
WAC 3′ UTR:
PUM 1 CLIP
63.53
0
PUM 2 CLIP
7.97
0
3505 bases
NGDN 3′ UTR:
PUM 1 CLIP
81.65
0
PUM 2 CLIP
0
0
624 bases
MYSM1 3′ UTR:
PUM 1 CLIP
74.24
0
PUM 2 CLIP
39.02
0
5257 bases
C
1 kb
ATP6V1G1 3′ UTR:
PUM 1 CLIP
5.33
0
PUM 2 CLIP
79.87
0
1128 bases
SPOPL 3′ UTR:
PUM 1 CLIP
3.52
0
PUM 2 CLIP
122.87
0
4154 bases
PNMA1 3′ UTR:
PUM 1 CLIP
4.59
0
PUM 2 CLIP
104.37
0
794 bases

Supplementary Figure 3: PUM1 and PUM2 display differential binding. **A)** Number of sites within 3′ UTRs occupied by PUM1 (light blue), PUM2 (dark blue), or both (black). **B)** Example 3′ UTRs containing sites with strong binding signal for PUM1 (light blue) but not PUM2 (dark blue). Peaks of interest are depicted with a black arrow. **C)** Example 3′ UTRs containing sites with strong binding signal for PUM2 (dark blue) but not PUM1 (light blue). Peaks of interest are depicted with a black arrow.

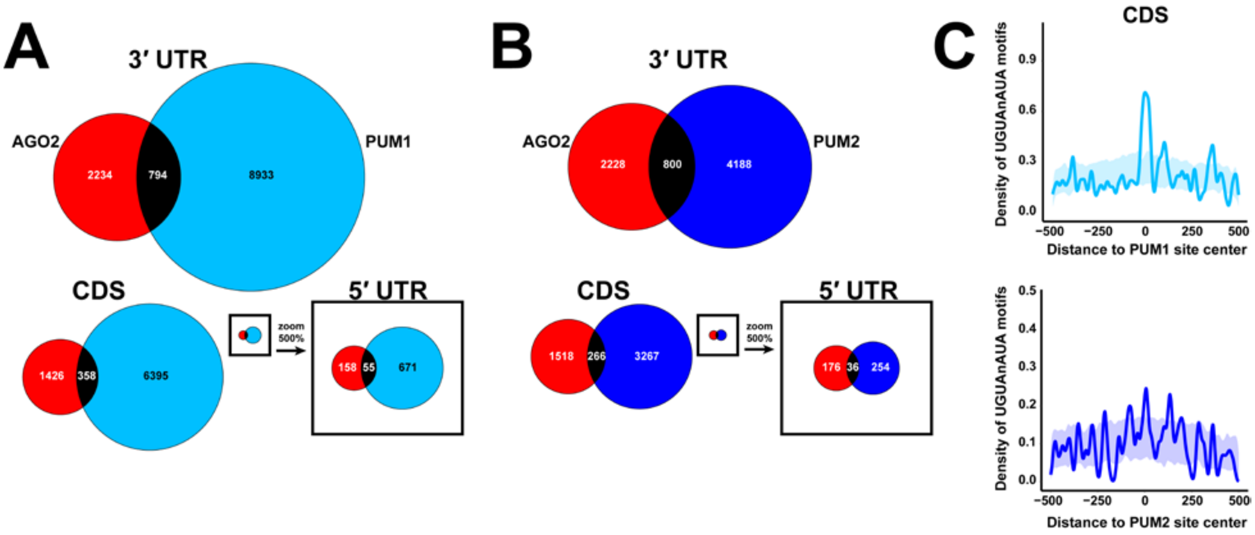

***Supplementary Figure 4: Venn Diagram of overlapping PUM and AGO2 sites*. A)** Number of sites within 3′ UTRs, the CDS, and 5′ UTRs occupied by AGO2 (red), PUM1 (light blue), or both (black). **B)** Number of sites within 3′ UTRs, the CDS, and 5′ UTRs occupied by AGO2 (red), PUM2 (dark blue), or both (black). **C)** Density of PUM motifs surrounding PUM1 (light blue) and PUM2 (dark blue) sites in CDS regions. The shaded areas represent randomized controls for the corresponding populations.

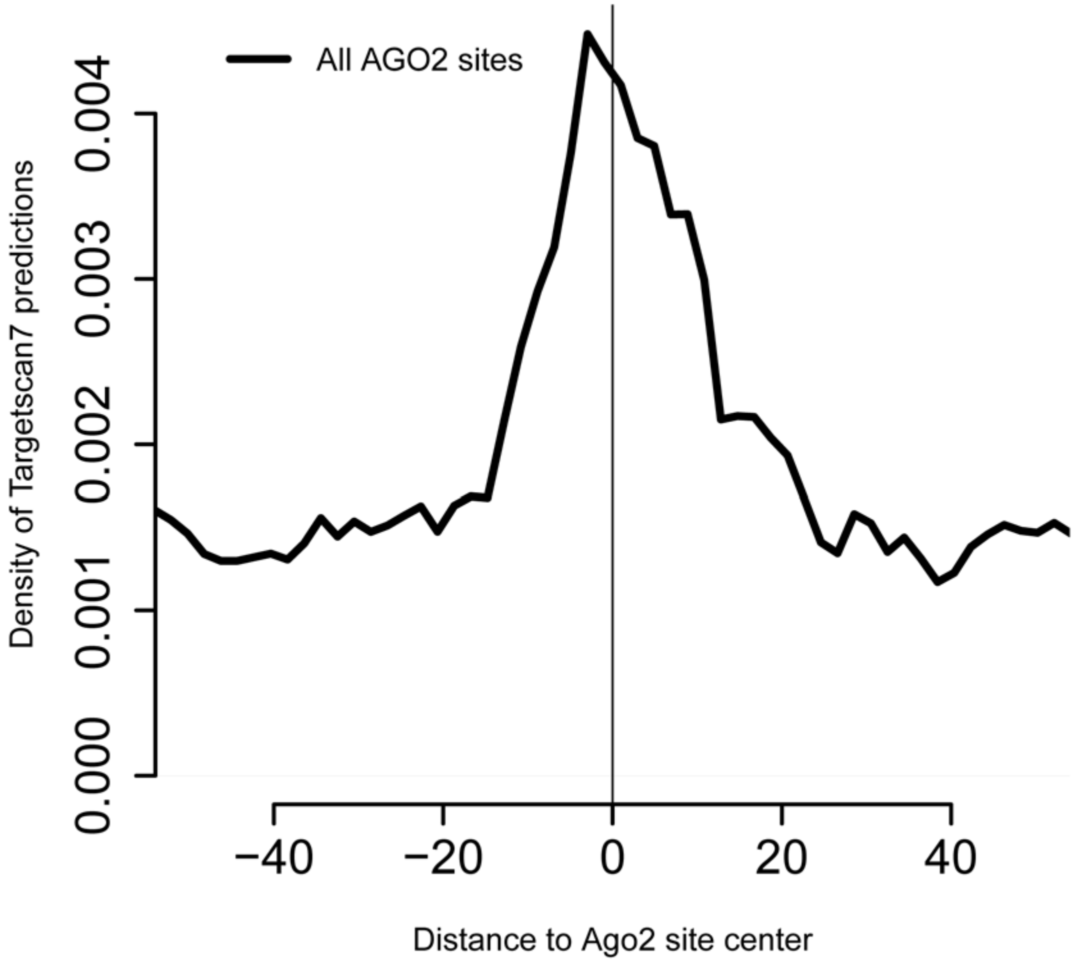

Supplementary Figure 5: AGO2 peak centers overlap with predicted miRNA seed sites. Density of Targetscan7 predictions surrounding all AGO2 peaks. A vertical black line denotes the AGO2 peak center.

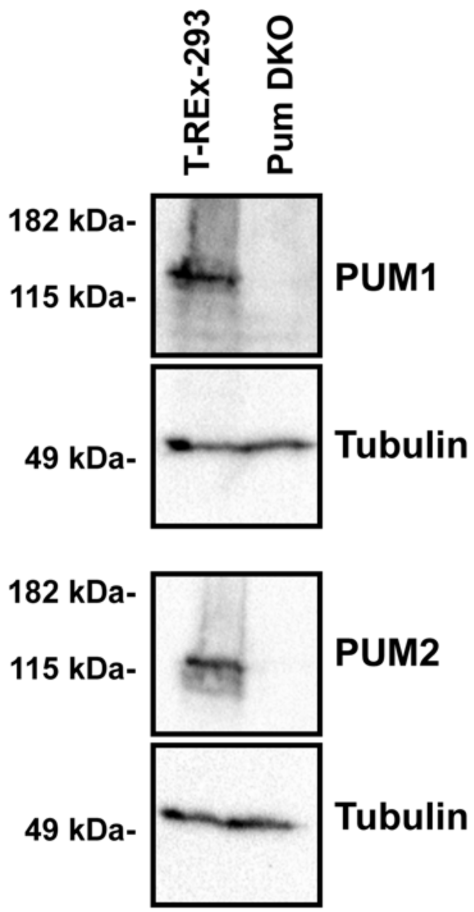

Supplementary Figure 6: Western blot of PUM1 and PUM2 in T-REx-293 and PUM double knockout cells. Two membranes were run in parallel using the same lysates and independently probed for PUM1 and PUM2. Each blot was then re-probed for Tubulin as a loading control. Full images can be found in Figure S12.

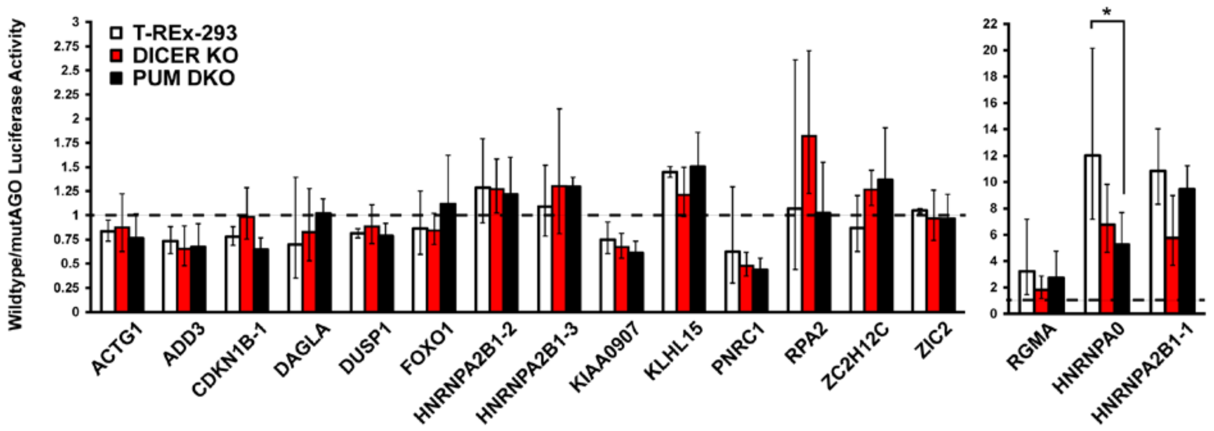

Supplementary Figure 7: Candidate sites that were not examined further. A subset of candidates showed no regulation in all cell conditions (ACTG1, DAGLA, FOXO1, HNRNPA2B-2, HNRNPA2B1-3, ZC3H12C, ZIC2), regulation that was not significantly dependent on AGO or PUM (ADD3, CDKN1B, DUSP1, KIA0907, KLHL15, PNRC1, RGMA, HNRNPA2B1-1), or excessively high protective site activity (HNRNPA0).

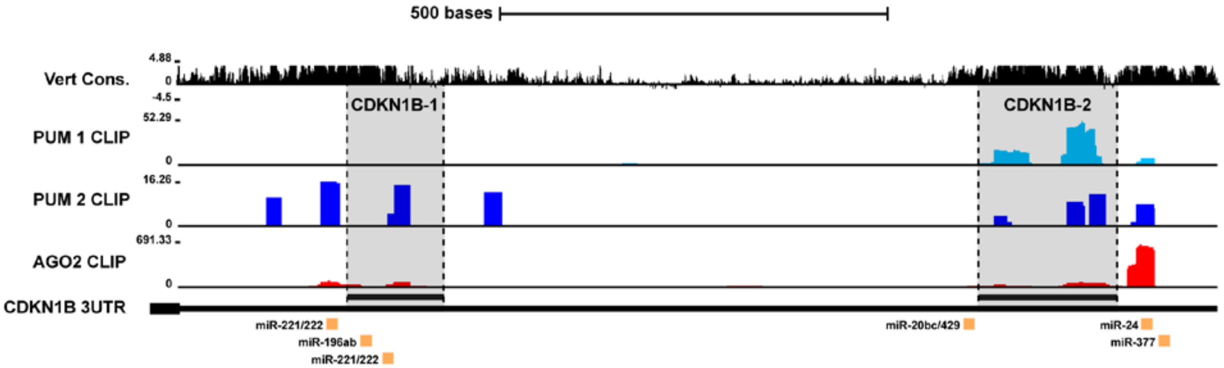

Supplementary Figure 8: UCSC browser view displaying the CDKN1B 3′ UTR. Tracks show the Vertebrate conservation score (black) as well as CLIP data for PUM1 (light blue), PUM2 (dark blue), and AGO2 (red). Candidate regions CDKN1B-1 and CDKN1B-2 are shaded gray.

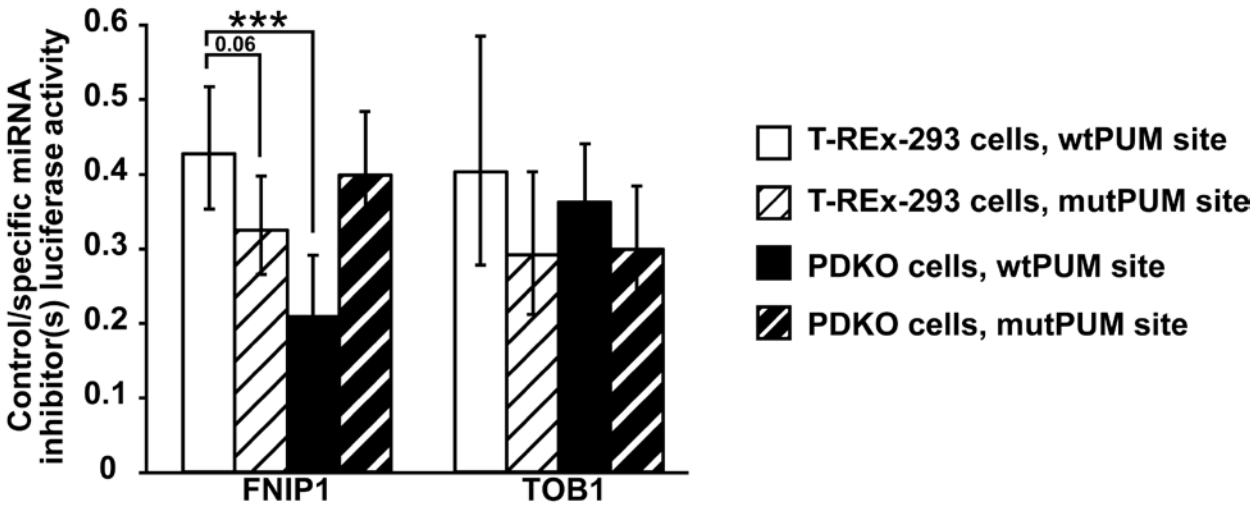

Supplementary Figure 9: Effects of PUM deletion or PUM site mutation on miRNA-dependent regulation of FNIP1 and TOB1 reporter constructs. Luciferase reporter constructs co-transfected with control or specific miRNA inhibitors were tested under wildtype (white) and PUM double knockout (black) conditions with WT (solid) or mutant PUM sites (stripes).

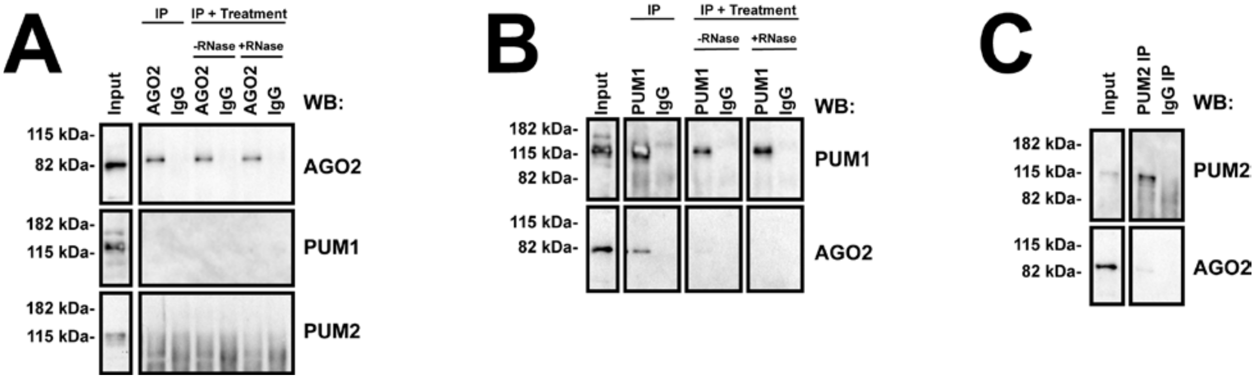

Supplementary Figure 10: Immunoprecipitation of AGO2, PUM1, and PUM2 shows evidence of weak interactions. **A)** AGO2 immunoprecipitation followed by AGO2, PUM1, and PUM2 immunoblot. Immunoprecipitated complexes were assayed immediately after washing (IP), or treated with PBS (IP + Treatment, -RNase) or 50µg/mL RNase A (IP + Treatment, +RNase) for an additional 20 minutes, followed by three washes. **B)** PUM1 immunoprecipitation followed by PUM1 and AGO2 immunoblot, treated as in (A). **C)** PUM2 immunoprecipitation followed by PUM2 and AGO2 immunoblot. Species-matched nonspecific IgG was used as a negative control. Each IP consists of one membrane probed for all relevant proteins. Full images can be found in Figure S12. T-Rex293 cells were cultured to 70% confluency in two 15 cm plates and lysed in hypotonic lysis buffer (HLB, 10 mM Tris-Cl at pH7.5, 10 mM KCl, 2 mM $MgCl_2$, 5 mM DTT, 1x protease inhibitor (Thermo cat #78441)). The cytoplasmic fraction was separated by centrifugation at 13,000 rpm for 30 minutes at 4°C and supplemented with 450mM KCl. 100 µl Protein A Dynabeads (Invitrogen, cat #2020-01-31) were incubated with 20 µg antibody on a rotator for 1 hour at room temperature. For the AGO2 IP, Protein A beads were bound first by a rabbit anti-mouse bridge (Jackson Immuno cat #315-005-003) followed by a mouse anti-AGO2 antibody (as described in methods). For the PUM1 IP, Protein A beads were bound first by a rabbit anti-goat bridge (Jackson Immuno cat#305-005-045) followed by a goat anti-PUM1 antibody (as described in methods). For the PUM2 IP, Protein A beads were directly bound with a rabbit anti-PUM2 antibody (as described in methods). Beads were incubated with cell lysate overnight at 4°C. Bead-protein complexes were washed

three times with HLB + 450 mM KCl. One-third of the beads were resuspended in 2xSDS loading buffer. The rest of the beads were incubated on rotator for 20 minutes at room temperature with either PBS or 50 µg/ml RNase A, washed three times with PBS, and resuspended in 2xSDS loading buffer. For AGO2 western blots, a second AGO2 antibody (Sigma cat#SAB420085) was used.

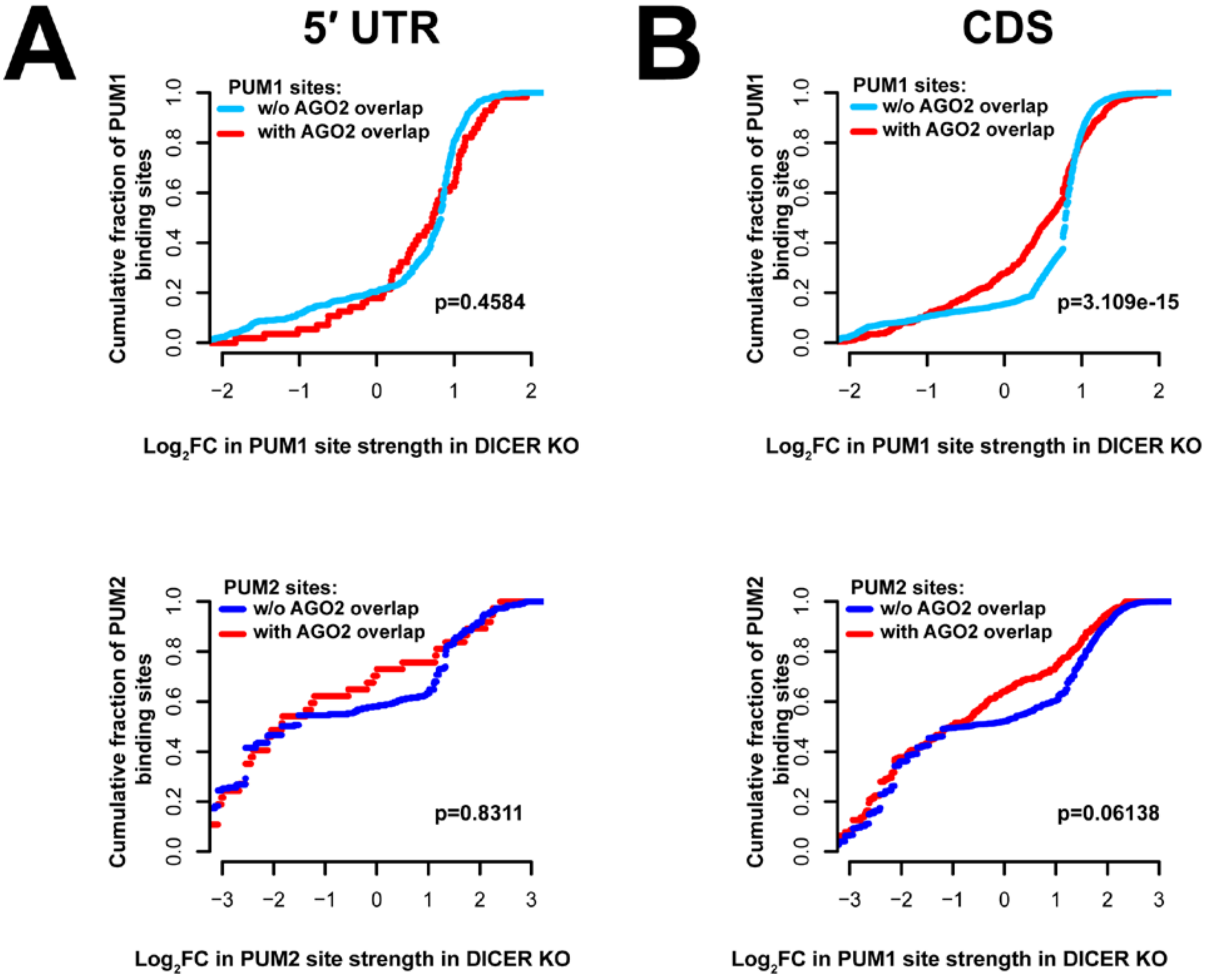

Supplementary Figure 11: AGO2-PUM site co-occupancy affects PUM1 binding in CDS sites, but not PUM2 binding in the CDS or PUM1 and PUM2 binding in the 5′ UTR. **A)** Cumulative distribution plots of the log fold change in PUM site strength, with AGO2 overlap (red) and without AGO2 overlap (PUM1: light blue, PUM2: dark blue) within 5′ UTRs. **B)** Cumulative distribution plots of the log fold change in PUM site strength, with AGO2 overlap (red) and without AGO2 overlap (PUM1: light blue, PUM2: dark blue) within the CDS.

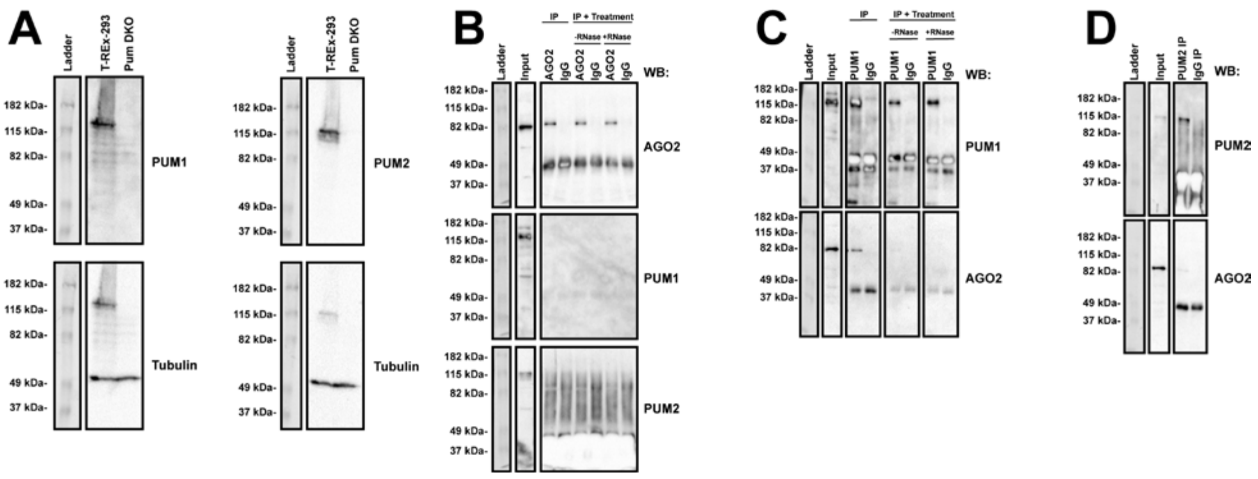

Supplementary Figure 12: Full length western blot images. **A)** Full length western blots of PUM1 and PUM2 in T-REx-293 and PUM double knockout cells. Cropped images and description shown in Figure S6. **B)** AGO2 immunoprecipitation followed by AGO2, PUM1, and PUM2 immunoblot. Cropped images and description shown in Figure S10A. **C)** PUM1 immunoprecipitation followed by PUM1 and AGO2 immunoblot. Cropped images and description shown in Figure S10B. **D)** PUM2 immunoprecipitation followed by PUM2 and AGO2 immunoblot. Cropped images and description shown in Figure S10C.

Supplementary File 1: AGO2 and PUM CLIP alignment and annotation information.

Supplementary File 2: Sequence information for siRNAs used for PUM1 and PUM2 knockdown, guide RNAs used for generation of PUM double knockout cells

Supplementary File 3: Sequence information for candidate sites and luciferase assay constructs.
